# Percutaneous left atrial appendage closure with concomitant dual-device implantation: a single-center observational study

**DOI:** 10.3389/fcvm.2025.1692521

**Published:** 2025-12-19

**Authors:** Antanas Gasys, Roberto Galea, Tommaso Bini, Juan Perich-Krsnik, Marco Gamardella, Laurent Roten, George C. M. Siontis, Bernhard Meier, Lorenz Räber

**Affiliations:** 1 Department of Cardiology, Bern University Hospital, University of Bern, Bern, Switzerland; 2 Department of Cardiology, Hospital Centre of Biel, Biel, Switzerland; 3 Graduate School for Health Sciences, University of Bern, Bern, Switzerland

**Keywords:** dual-device, left atrial appendage closure, multilobed anatomy, pericarditis, peridevice leak

## Abstract

**Background:**

Percutaneous left atrial appendage (LAA) closure (LAAC) is a proven stroke prevention strategy for patients with atrial fibrillation (AF). However, incomplete sealing in complex LAA anatomies may compromise efficacy.

**Objectives:**

This study evaluates the safety, feasibility, and efficacy of concomitant dual-device LAAC in multilobed anatomies, representing the largest cohort examined to date.

**Methods:**

We reviewed all LAAC procedures performed at the University Hospital of Bern between 2009 and 2025. Baseline characteristics, procedural details, and follow-up data were analyzed for patients receiving dual-device LAAC. Endpoints included technical success, complications, thromboembolic events, and device-related issues. Continuous data were expressed as mean ± standard deviation or median values, while categorical data were reported as percentages. Group comparisons were conducted using *t*-test, Mann–Whitney *U* test, or chi-square test. Differences were expressed as 95% confidence intervals, and a *p*-value of less than 0.05 was considered significant.

**Results:**

Of 1,307 LAAC procedures, 10 included dual-device implantation. The mean age of the patients was 71 years, and all patients were men. The Congestive heart failure, Hypertension, Age ≥75 years, Diabetes mellitus, prior Stroke or TIA, Vascular disease, Age 65–74 years, Sex category (CHA_2_DS_2_-VASc) and Hypertension, Abnormal renal/liver function, Stroke, Bleeding history or predisposition, Labile INR, Elderly, Drugs/alcohol (HAS-BLED) scores were 3.5 ± 1.8 and 2.9 ± 1.4, respectively. Most patients (70%) had paroxysmal AF. Preprocedural and intraprocedural transesophageal echocardiography (TEE) confirmed multilobed LAA anatomy in all cases. Half of the procedures were fluoroscopy-guided with one delivery sheath and transseptal puncture, while the other half were TEE-guided with a double sheath and two transseptal punctures. Only Amplatzer devices were used: Eight procedures employed two devices of the same type [five Amulet, three Amplatzer Cardiac Plug (ACP)], and two procedures combined different types (Amulet + ACP, ACP + Amplatzer Vascular Plug). Technical success was achieved in all cases. Within the first week, one (10%) patient experienced a clinically non-clinically relevant pericardial effusion. At 1-year follow-up (completed in nine patients), three (30%) patients developed pericarditis. No thromboembolic events, device-related thrombosis, or device embolization occurred.

**Conclusion:**

In this small cohort of patients with complex multilobed LAA anatomy, concomitant implantation of two Amplatzer devices proved to be a feasible strategy with acceptable short-term safety, although potentially associated with an increased risk of pericarditis.

## 1 Introduction

Percutaneous left atrial appendage (LAA) closure (LAAC) is a valuable therapeutic option for stroke prevention in patients with atrial fibrillation (AF) who have contraindications to long-term oral anticoagulation (1). The ultimate goal of the LAAC procedure is to accomplish a complete seal of the LAA ostium, thereby excluding the LAA from circulation, which is the main cardiac source of thrombi (2). However, due to its heterogeneous anatomy, complete percutaneous LAA sealing is not always possible with the currently available devices, leading to either aborted procedures or clinically relevant residual leaks (3, 4).

A large multilobed LAA represents one of the most challenging LAA anatomies for percutaneous closure. The large ostia associated with proximal internal septa between lobes often complicate device implantation, as there is no sufficient depth to accommodate a large device proximally or the disc of a device implanted in the larger lobe may not cover the ostium entirely. Alternative strategies, including the implantation of two LAAC devices, have been proposed (5–10). However, studies reporting two-device implantation are limited by small sample sizes, lack of documentation of device-related complications, and the absence of long-term clinical outcomes. Therefore, further investigation is warranted.

This retrospective observational study evaluated procedural success, safety, and clinical outcomes up to 1 year—including cerebrovascular events, bleeding, and device-related complications—following concomitant implantation of two LAAC devices.

## 2 Methods

### 2.1 Study population

This study included all consecutive patients who underwent attempted LAAC at Bern University Hospital between January 2009 and January 2025. It combines data from two cohorts: a historical cohort of prospectively collected procedures (January 2009–July 2015) that was previously described (11) and a prospective registry of consecutive procedures initiated in August 2015 (NCT04628078). There were no formal exclusion criteria; all patients who provided informed consent were included. Patients were classified into the “single-device group” if they received a single LAAC device and the “dual-device group” if they received multiple devices during the procedure. The study was conducted in accordance with the Declaration of Helsinki and received approval from the institutional ethics committee.

### 2.2 LAAC procedure

**Figure 1:**
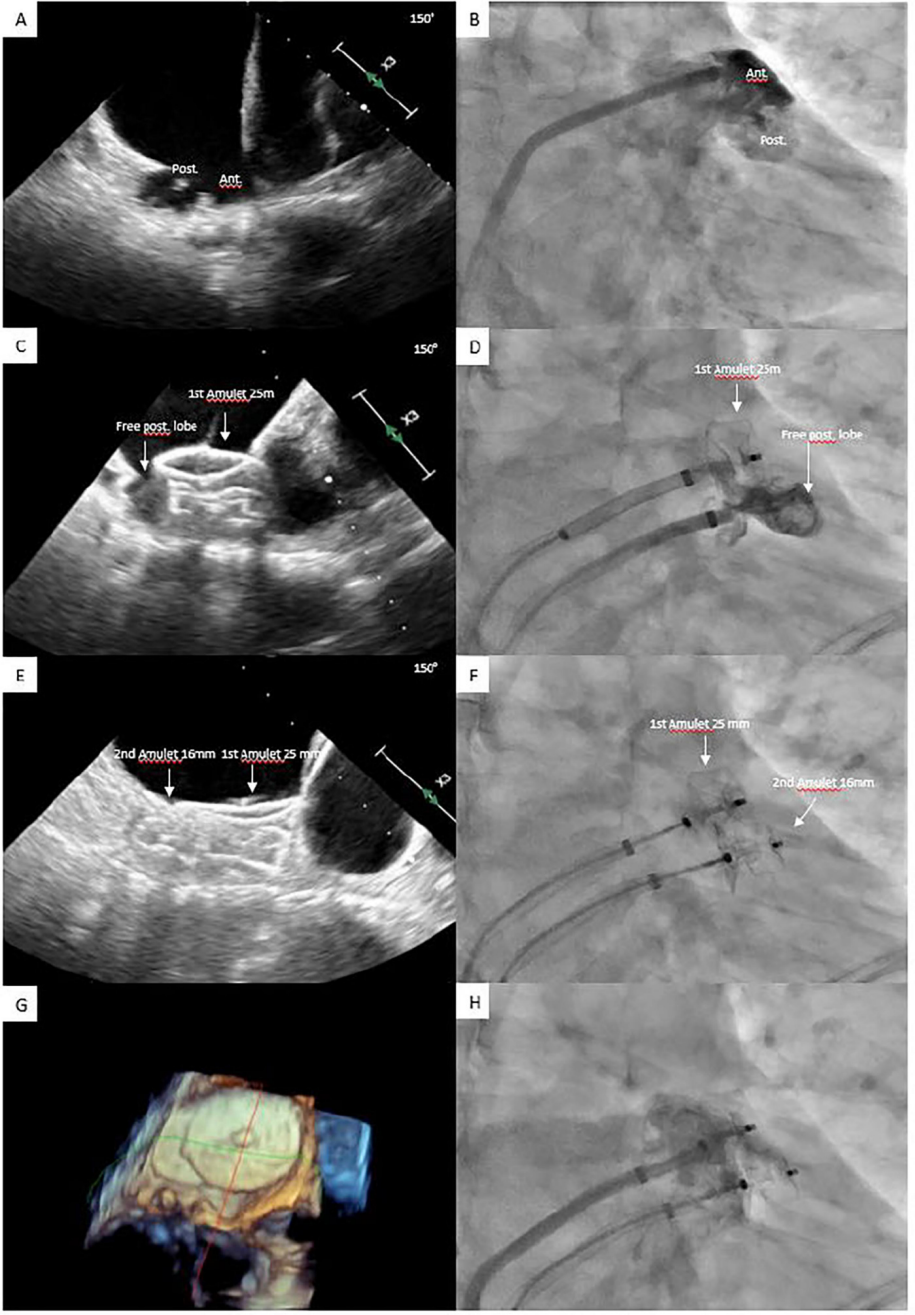
Percutaneous LAAC with concomitant implantation of two LAAC devices by using the **double-sheath technique**. Large bilobed LAA showed by TEE **(A)** and angiography **(B)**. Residual patent lobe after implantation of Amulet 25 mm showed by TEE **(C)** and angiography **(D)**. Complete sealing of bilobed LAA after implantation of a second Amulet device **(E–H)**. LAA, left atrial appendage; LAAC, left atrial appendage closure; TEE, Transesophageal echocardiography.

**Figure 2:**
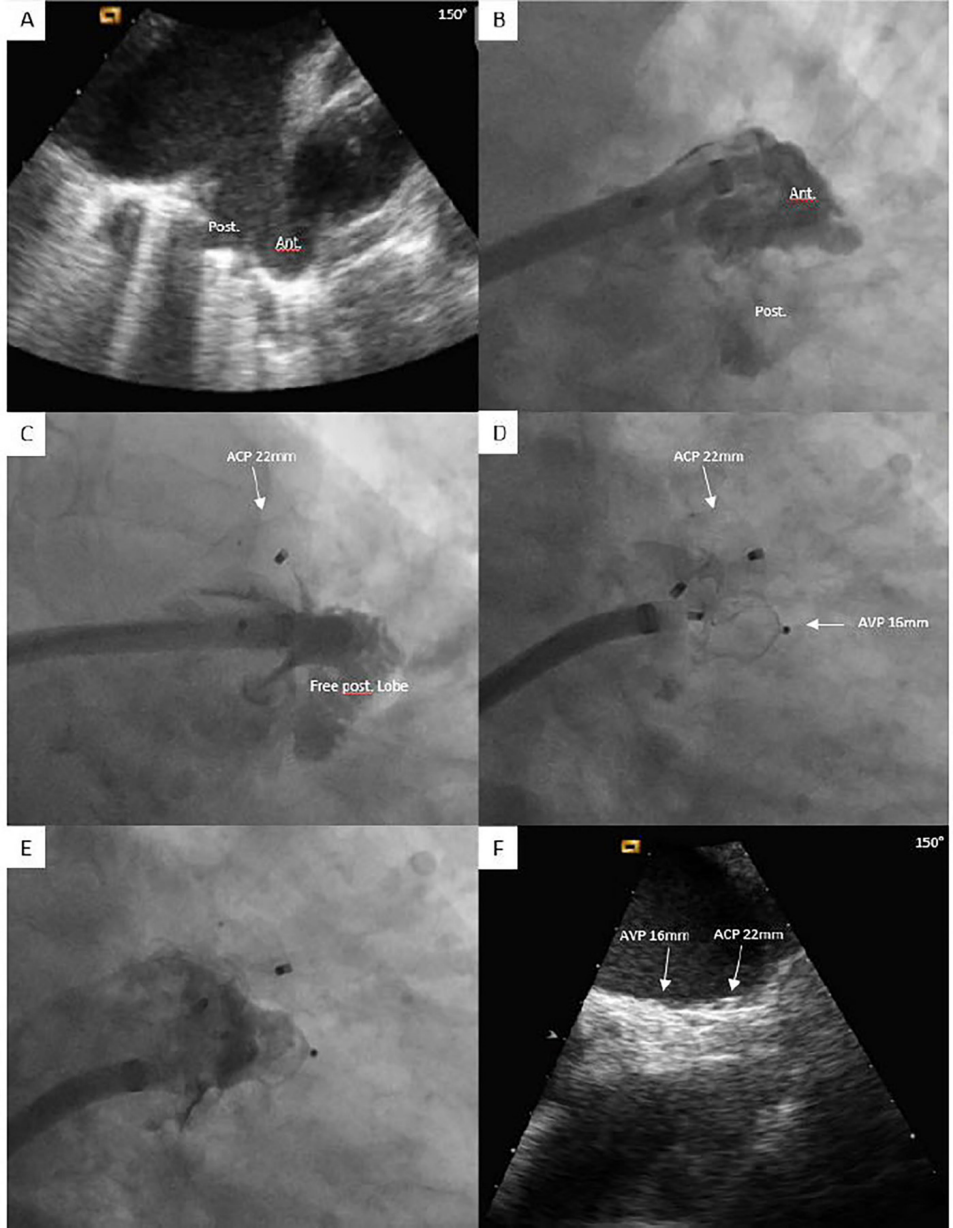
Percutaneous LAAC with concomitant implantation of two LAAC devices by using the **single-sheath technique**. Large bilobed LAA with anterior and posterior lobe showed by TEE **(A)** and angiography **(B)**. Residual patent posterior lobe after implantation of ACP showed by angiography **(C)**. Complete sealing of bilobed LAA after implantation of AVP 16 mm at the end of procedure by angiography and during the follow-up by TEE **(D–F)**. ACP, Amplatzer Cardiac plug; AVP, Amplatzer Vascular plug; LAA, left atrial appendage; LAAC, left atrial appendage closure; TEE, Transesophageal echocardiography.

Preprocedural and intraprocedural transesophageal echocardiography (TEE) was typically performed to rule out LAA thrombus. LAAC procedures followed institutional practice (12), beginning with transvenous femoral access and transseptal puncture to enter the left atrium. In the single-device group, two types of LAAC devices were used: single-closure system devices (Watchman or Watchman FLX, Boston Scientific, MA, USA) and pacifier-principle plug-and-disc devices [Amplatzer Cardiac Plug (ACP) or Amulet, St. Jude Medical/Abbott, IL, USA]. In the dual-device group, only Amplatzer devices [ACP, Amulet, or Amplatzer Vascular Plug (AVP)] were implanted. A device-specific sheath was advanced over a stiff 0.035-inch guidewire into the left atrium and positioned in the proximal LAA. LAA angiography was performed in multiple projections, including right anterior oblique caudal, cranial, and lateral views. The procedure was guided by fluoroscopy, with or without TEE. Device size was determined using LAA angiography, preprocedural and intraprocedural TEE, or computer tomography. Following a sustained tug test, stability and LAA sealing were assessed via angiography and/or TEE before device release. In the event of uncovered LAA lobes or large residual peridevice leak (PDL), the implantation of a second device was attempted. For second device implantation, either a “single-sheath” or a “double-sheath technique” was used (Figures 1, 2). “The single-sheath technique” involved releasing the first device and implanting the second using the same sheath, with no additional transseptal puncture (Figure 2). “The double-sheath technique” required an additional venous and transseptal puncture, using a second sheath, with both devices released at the end of the procedure (Figure 1). The second device was selected by the operator based on angiographic and/or TEE measurements, considering the morphology and dimensions of the uncovered area. Post-LAAC antithrombotic therapy was determined by the operator based on the patient's ischemic and bleeding risk (13, 14). Typically, dual antiplatelet therapy (DAPT) was prescribed for a period of 1–3 months followed by single antiplatelet therapy (SAPT). For patients with very high bleeding risk, SAPT was prescribed at discharge instead of DAPT (15).

**CENTRAL ILLUSTRATION:**
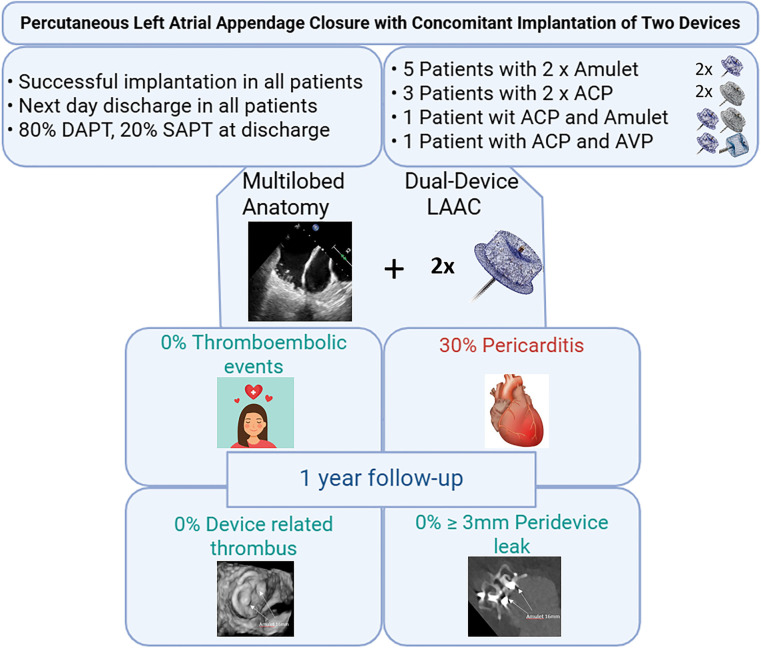
Main study results. ACP, Amplatzer Cardiac plug; AVP, Amplatzer Vascular plug; LAAC, left atrial appendage closure; DAPT, dual antiplatelet therapy; SAPT, single antiplatelet therapy.

### 2.3 Follow-up

After the procedure, all patients underwent TEE prior to discharge to exclude device embolization and pericardial effusion. Follow-up was conducted using a standardized questionnaire, phone interviews, or outpatient visits. All adverse events were documented, including death, cerebrovascular and thromboembolic events, and bleeding episodes, which were classified according to the Bleeding Academic Research Consortium (BARC) classification.

Acute pericarditis was defined by the presence of at least two of the following: typical chest pain, pericardial friction rub, new diffuse ST-segment elevation or PR-segment depression, and new or worsening pericardial effusion. New-onset pericardial effusion was categorized as clinically relevant (associated with tamponade physiology, low cardiac output, pulsus paradoxus, or requiring intervention or surgery) or non-clinically relevant (asymptomatic). Vascular access complications were also captured.

Adverse events were classified and adjudicated by a clinical event committee consisting of two cardiologists, with a third cardiologist in case of disagreement. Cerebrovascular events were reviewed and adjudicated by a neurologist.

Routine TEE follow-up was performed 45–90 days after LAAC. Device-related complications included device-related thrombus (DRT), defined as an echodensity on the left atrial device surface not attributable to artifact, inconsistent with normal healing or device incorporation, visible in multiple TEE planes, in contact with the device, and demonstrating independent motion (16). PDLs were classified on color Doppler as <3 or ≥3 mm residual flow between the left atrium and the LAA, indicating persistent LAA patency. PDL was assessed based on the single largest jet visualized around the device and considered significant if the diameter was ≥3 mm (16).

### 2.4 Study endpoints

The study endpoints included technical success, defined as complete LAA sealing (no PDL >5 mm on color Doppler TEE or angiography), complete exclusion of the LAA, and the absence of device-related complications at the end of the procedure (17); clinical events at 7 and 365 days following the procedure; and device-related complications at TEE follow-up including DRT and PDL (<3 or ≥3 mm). Clinical events included overall death, cerebrovascular events, systemic embolism, bleeding (BARC 2 or BARC 3–5), acute pericarditis, pericardial effusion (clinically relevant or non-clinically relevant), vascular access complications, and device embolization.

### 2.5 Statistical analysis

Continuous variables are summarized as mean ± standard deviation (SD), and categorical variables as counts and percentages. Variables were compared using Student's t-tests, Mann–Whitney tests, or chi-square tests as appropriate. Differences in proportions and means were expressed with 95% confidence intervals. All tests were two-sided, and a *p*-value of less than 0.05 was considered statistically significant. Statistical analyses were conducted using IBM SPSS Statistics, version 25 (IBM Corp, NY, USA).

## 3 Results

A total of 1,307 consecutive LAACs were performed at the University Hospital of Bern (Switzerland) between January 2009 and January 2025. In 1,297 cases (99.2%), a conventional, single-device technique was used, and in 10 cases (0.8%), a dual-device technique was employed (Central Illustration). Baseline characteristics are summarized in Table 1. All patients in the dual-device group were men. The mean Congestive heart failure, Hypertension, Age ≥75 years, Diabetes mellitus, prior Stroke or TIA, Vascular disease, Age 65–74 years, Sex category (CHA_2_DS_2_-VASc) and Hypertension, Abnormal renal/liver function, Stroke, Bleeding history or predisposition, Labile INR, Elderly, Drugs/alcohol (HAS-BLED) scores were 3.5 ± 1.8 and 2.9 ± 1.4, respectively. Paroxysmal AF was present in 70% of the cases and a history of stroke was present in 10% of patients in the dual-device group.

**Table 1:** Baseline characteristics.

Patient related characteristics	Dual-device group (*N* = 10)	Single-device group (*N* = 1,297)	Difference and 95% confidence interval	*p*-value
Age (years), mean ± SD	71.9 ± 8.9	78.5 ± 93.8	−6.6 (−64.8 to 51.7)	0.825
Female, *n* (%)	0 (0.0%)	376 (29%)	−29.0% (−57.2% to 0.8%)	0.071
CHA_2_DS_2_-Vasc score, mean ± SD	3.5 ± 1.8	4.3 ± 1.6	−0.8 (−1.8 to 0.2)	0.129
HAS-BLED score, mean ± SD	2.9 ± 1.4	3.1 ± 1.0	−0.2 (−0.8 to 0.4)	0.477
Paroxysmal AF, *n* (%)	7 (70%)	712 (55%)	15.1% (−15.9% to 6.1%)	0.526
Arterial hypertension, *n* (%)	8 (80%)	1,084 (84%)	−3.6% (−26.7% to 19.5%)	0.673
Diabetes mellitus, *n* (%)	2 (20%)	331 (26%)	−5.5% (−32.7% to 21.6%)	1
Chronic kidney disease^a^, *n* (%)	1 (10%)	150 (12%)	−1.6% (−21.5% to 18.4%)	1
Prior cerebrovascular event, *n* (%)	1 (10%)	399 (31%)	−20.8% (−49.5% to 0.9%)	0.299
Systemic embolism, *n* (%)	1 (10%)	40 (3%)	6.9% (−3.9% to 17.8%)	0.274
Known CAD, *n* (%)	5 (50%)	657 (51%)	−0.7% (−31.8% to 30.5%)	1
Vascular disease^b^, *n* (%)	5 (50%)	638 (50%)	0.8% (−30.3% to 2.0%)	1
History of relevant bleeding, *n* (%)	7 (70%)	773 (60%)	10.4% (−20.2% to 1.0%)	0.748
LVEF (%), mean ± SD	53.5 ± 6.3	54.9 ± 11.1	−1.4 (−8.3 to 5.5)	0.689

AF, atrial fibrillation; CAD, coronary heart disease; LVEF, left ventricular ejection fraction; TEE, transesophageal echocardiography.

a Chronic kidney disease is defined if at least one of the following criteria is met: estimated glomerular filtration rate 200 mmol/L, and dialysis or history of kidney transplantation.

b Vascular disease was defined as the presence of any of the following: history of myocardial infarction, intermittent claudication, previous surgery or percutaneous intervention on the abdominal aorta or the lower extremity vessels, abdominal or thoracic surgery, and arterial and venous thrombosis.

### 3.1 LAAC procedures

Procedural characteristics are summarized in Table 2. Imaging assessments of LAAs using TEE and angiography identified a complex, multilobed anatomy in all cases (100%). Both the LAA ostium and landing zone were significantly larger in the dual-device group compared to the single-device group in both TEE (32.3 vs. 24.2 mm; *p* < 0.001 and 23.8 vs. 19.0 mm; *p* = 0.002) and LAA angiography (31.3 vs. 26.4 mm; *p* = 0.014 and 25.5 vs. 21.4 mm; *p* = 0.008).

**Table 2:** Imaging and procedural characteristics.

Procedural characteristics	Imaging characteristics	Dual-device group (*N* = 10)	Single-device group (*N* = 1,297)	Difference and 95% confidence interval	*p*-value
Transesophageal echocardiography evaluation	LAA ostium, maximum diameter (mm), mean ± SD	*n* = 10, 32.3 ± 9	*n* = 1,104, 24.2 ± 6	8.2 (4.2 to 12.1)	<0.001
	Landing zone, maximum diameter (mm), mean ± SD	*n* = 10, 23.8 ± 8	*n* = 1,100, 19.0 ± 5	4.8 (1.8 to 7.8)	0.002
	Depth (mm), mean ± SD	*n* = 10, 27.7 ± 6	*n* = 1,104, 26.1 ± 7	1.7 (−2.5 to 5.8)	0.431
	Distance from ostium to proximal septum (mm), mean ± SD	*n* = 10, 7.6 ± 1.9	NA	NA	NA
LAA angiography evaluation	LAA ostium^a^, maximum diameter (mm), mean ± SD	*n* = 9, 31.3 ± 9	*n* = 881, 26.4 ± 6	4.9 (1.0 to 8.8)	0.014
	Landing zone^b^, maximum diameter (mm), mean ± SD	*n* = 9, 25.5 ± 8	*n* = 882, 21.4 ± 5	4.2 (1.1 to 7.2)	0.008
	Depth^c^ (mm), mean ± SD	*n* = 9, 26.2 ± 10	*n* = 881, 31.0 ± 8	−4.8 (−10.0 to 0.4)	0.068
TEE guidance, *n* (%)		*n* = 5, (50%)	*n* = 672 (59%)	−14% (−47% to 18%)	0.500
General anesthesia, *n* (%)		*n* = 0, 0 (0%)	*n* = 1,297, 148 (11%)	−11% (−31% to 8%)	0.615
Device implantation attempts (*n*), mean ± SD		*n* = 10, 1.9 ± 2	*n* = 1,297, 1.6 ± 1	0.3 (−0.5 to 1.2)	0.431
Fluoroscopy time (min), mean ± SD		*n* = 10, 22.2 ± 13	*n* = 1,197, 15.5 ± 11	6.7 (−0.4 to 13.8)	0.064
Contrast medium (mL), mean ± SD		*n* = 10, 229.7 ± 163	*n* = 1,212, 133.5 ± 111	96.2 (26.6 to 165.8)	0.007

LAA, left atrial appendage; SD, standard deviation.

a Defined as a line measured from the coronary artery marker to the tip of the Coumadin ridge in at two least views.

b Defined as a line measured 10–12 mm distal to the orifice in at least two views and perpendicular to the main LAA axis.

c Defined as a line measured in at least two views from the orifice to the opposite wall in the expected device's axis.

A significantly higher amount of contrast medium was used during the dual-device procedure compared to the single-device approach (229.7 vs. 133.5 ml; *p* = 0.007). Other procedural characteristics did not significantly differ between the two groups, although fluoroscopy time (22.2 vs. 15.5 min; *p* = 0.064) and the number of device implantation attempts (1.9 vs. 1.6; *p* = 0.431) were numerically higher in the dual-device group. The distance from the main ostium to the commencement of the proximal septum in the dual-device group yielded a mean distance of 7.6 ± 1.9 mm.

Specific characteristics of the 10 dual-device procedures are reported in Table 3. Indications for LAAC in dual-device group included previous bleeding in seven patients, intolerance to anticoagulation in one patient, breakthrough stroke in one patient, and high bleeding risk due to terminal renal insufficiency and coronary artery disease with prior stenting requiring dual antiplatelet therapy to avoid triple therapy in one patient. Five of 10 cases were performed under fluoroscopy-only guidance and with a single-sheath technique (i.e., one transseptal puncture) (Figure 2). Of them, in three cases, two ACPs of different sizes were used, whereas one patient received a combination of an ACP and AVP, while another had an Amulet paired with an ACP. The remaining five procedures were performed under TEE guidance using a double-sheath technique (i.e., two transseptal punctures) (Figure 1). In these five cases, two Amulets were implanted.

**Table 3:** Characteristics of the dual-device group interventions.

Patient	LAAC indication	Imaging guidance (fluoroscopy only vs. TEE)	Closure approach (single vs. double sheath)	First device implanted (type/size)	Second device implanted (type/size)	Discharge antithrombotic therapy
No. 1	Previous bleeding	Fluoroscopy only	Single	ACP 22 mm	AVP 16 mm	1 month DAPT followed by SAPT with aspirin
No. 2	Anticoagulation intolerance	Fluoroscopy only	Single	ACP 20 mm	ACP 26 mm	3 months DAPT followed by SAPT with aspirin
No. 3	Previous bleeding	Fluoroscopy only	Single	ACP 30 mm	ACP 30 mm	3 months DAPT followed by SAPT with aspirin
No. 4	Breakthrough stroke	Fluoroscopy only	Single	ACP 30 mm	ACP 16 mm	5 months DAPT followed by SAPT with aspirin
No. 5	Previous bleeding	Fluoroscopy only	Single	Amulet 20 mm	ACP 28 mm	3 months DAPT followed by SAPT with aspirin
No. 6	Previous bleeding	TEE	Double	Amulet 16 mm	Amulet 16 mm	6 months SAPT with aspirin
No. 7	High bleeding risk^a^	TEE	Double	Amulet 18 mm	Amulet 16 mm	2 months DAPT followed by SAPT with aspirin
No. 8	Previous bleeding	TEE	Double	Amulet 16 mm	Amulet 16 mm	3 months DAPT followed by SAPT with aspirin
No. 9	Previous bleeding	TEE	Double	Amulet 20 mm	Amulet 16 mm	12 months SAPT with aspirin
No. 10	Previous bleeding	TEE	Double	Amulet 25 mm	Amulet 16 mm	1 month DAPT followed by SAPT with aspirin

ACP, Amplatzer cardiac plug; AVP, Amplatzer vascular plug; DAPT, dual antiplatelet therapy; LAAC, left atrial appendage closure; SAPT, single antiplatelet therapy; TEE, transesophageal echocardiography.

a Terminal renal insufficiency and coronary artery disease with prior stenting, requiring dual antiplatelet therapy to avoid triple therapy.

Eight of 10 patients were discharged under DAPT for at least 1 month followed by SAPT, whereas the two remaining patients were discharged under SAPT (Table 3).

### 3.2 Study endpoints

Technical success was achieved in all dual-device procedures with no residual PDL or patent lobe detected at the end of procedure. All 10 patients were discharged the day following the procedure. At 7-day follow-up, one patient (10%) experienced a minor vascular access complication that required no treatment, and another developed acute pericarditis (double-sheath technique; Amulet 20 and 16 mm) after LAAC, characterized by typical chest pain and a small pericardial effusion, which was treated with colchicine and resolved without sequelae. No further procedural complications were reported. One-year follow-up (mean 391 ± 62.9 days) was available in 9 out of 10 patients, since one patient had undergone the procedure only 8 months previously. During this time, two patients developed acute pericarditis at 10 days (double-sheath technique; 2× Amulet 16 mm) and 11 months (single-sheath technique; ACP 22 mm and AVP 16 mm) following LAAC, respectively. Both presented with characteristic chest pain, electrocardiographic changes, and small pericardial effusions. Management was conservative—aspirin and colchicine in one case, aspirin alone in the other—with complete resolution and no sequelae. Furthermore, gastrointestinal bleeding occurred in two patients: one classified as BARC 2 at 8 months and another classified as BARC 3a at 10 months following the procedure. No death, stroke, thromboembolic events, or device embolization was reported within 12 months after LAAC (Table 4).

**Table 4:** Adverse events at 7 days and 1 year after the procedure in the dual-device group.

Adverse events	At 7 days after procedure (*n* = 10)	Between 7 and 365 days after procedure (*n* = 9^a^)
All-cause death, *n* (%)	0 (0%)	0 (0%)
Cerebrovascular event, *n* (%)	0 (0%)	0 (0%)
Systemic embolism, *n* (%)	0 (0%)	0 (0%)
Minor (BARC 2), *n* (%)	0 (0%)	1 (11%)
Major (BARC 3–5), *n* (%)	0 (0%)	1 (11%)
Acute pericarditis, *n* (%)	1 (10%)	2 (22%)
Pericardial effusion		
- Clinically relevant	0 (0%)	1 (11%)
- Non-clinically relevant	1 (10%)	0 (0%)
Vascular access-related complication, *n* (%)	1 (10%)	0 (0%)
Device embolization, *n* (%)	0 (0%)	0 (0%)
Device-related thrombus *n* (%)	0 (0%)	
Peridevice leak in TEE		
- <3 mm *n* (%)	3 (30%)	
- ≥3 mm *n* (%)	0 (0%)	

BARC, Bleeding Academic Research Consortium; TEE, transesophageal echocardiography.

a 1 year follow-up is available in nine patients, since one procedure was performed during the last 12 months.

At 45-day TEE follow-up, which was available for all 10 patients, no DRT was reported. Three patients had a minor PDL <3 mm with no therapeutic consequence.

## 4 Discussion

From the groundbreaking PREVAIL and PROTECT AF studies to the advancements highlighted in the OPTION trial, percutaneous LAAC has steadily evolved into a safer and more effective therapy for AF patients over the past decade (16, 17). This progress has been driven by factors such as the increased use of preprocedural and intraprocedural imaging, and improvements in device design, both of which have contributed to a gradual increase in procedural technical success (11, 18). However, despite these advancements, the complexity of anatomical variations of the LAA remains a significant challenge, as not all anatomies can be successfully sealed percutaneously with the currently available devices (1, 19).

Multilobed, large LAAs present technical challenges, often hindering the ability to achieve complete sealing with only one device. The key driver of complexity in multilobed anatomies is an internal septum that reduces the working depth to a degree that no longer allows for a proximal device implantation. The mean maximum proximal landing zone dimensions in our dual-device cohort were 23.8 mm on TEE and 25.5 mm on angiography, both within the feasible range to accommodate a single device. However, with an average distance of 7.6 mm between the intralobar septum and the ostium, proximal device implantation was not feasible, as a minimum working depth of 10 mm is required for proper deployment. Careful preprocedural and intraprocedural anatomical assessment is crucial for detecting multilobed anatomy and selecting the most appropriate treatment strategy, including the use of two devices (11).

To achieve full sealing in a complex multilobed anatomy, the procedural complexity increases. Accordingly, we noted a statistically significant increase in the amount of contrast medium used in the dual-device cohort (229.7 vs. 133.5 mL; *p* = 0.007). There was also a numerical increase in fluoroscopy time (22.2 vs. 15.5 min; *p* = 0.064) and the number of device implantation attempts (1.9 vs. 1.6; *p* = 0.431).

The existing evidence on dual-device implantation strategies consists of case reports and series. One report involved five patients treated with two Amplatzer devices (ACP, AVP, and Amplatzer septal occluder), while two small observational follow-up studies, each with seven cases, all used the Watchman device (5–10). To the best of our knowledge, this is the first observational study to analyze patients undergoing two concomitantly implanted Amplatzer devices, including five individuals treated with a combination of two Amulet devices—a strategy that has not been previously reported and, to the best of our knowledge, is not performed in clinical routine.

Upon compared with prior case series involving Amplatzer devices, which included two patients who underwent staged procedures with two devices and three patients who received all-in-one procedures, all patients in the present study were treated with two devices in a single intervention (5). In addition, in our study, five cases (50%) utilized a novel double-sheath approach, whereas all procedures in the earlier case series were performed using a single-sheath approach. In contrast to our study, the duration of follow-up in the previous case series was not specified, and critical adverse events, such as pericardial effusion, bleeding, and device-related complications (including PDLs or DRTs), were not systematically documented.

Regarding prior observational studies involving two Watchman devices, only one of the 14 patients underwent LAAC using a double-sheath approach, while the remaining patients were treated with a single-sheath approach.

The incidence of PDLs in the referenced cohorts with Watchman devices was documented at 28.6% and 14.2% (in all cases 2 mm), respectively (6, 10). In contrast, within our cohort treated with the Amplatzer device, no PDLs were observed at the end of the procedure. During the follow-up period, PDLs measuring <3 mm were observed in three patients (30%), all of which were deemed clinically insignificant. These PDLs were localized between the device and the Coumadin ridge, the device and the mitral valve annulus, and between devices, respectively. Traditionally, leaks exceeding 5 mm have been classified as clinically significant; however, emerging evidence suggests that even smaller leaks, ≥3 mm, may contribute to adverse clinical outcomes (20–22). The avoidance of any PDLs ≥3 mm, despite complex multilobed anatomy with large ostia, is notable, attesting to the high clinical efficacy of the dual-device closure technique reported here. Alternatives addressing the challenges of multilobed anatomies include the use of devices with much larger disc sizes (i.e., up to 36/40 mm), such as the LAmbre device (23). However, whether this approach reduces the incidence of PDLs in patients with extensive and multilobed LAA anatomy remains an unresolved question requiring further investigation.

Device-related complications following LAAC, such as DRT, may carry a higher risk with a dual-device approach due to the increased presence of foreign material. The additional device surface can expand the thrombogenic area and potentially delay endothelialization, further contributing to thrombus formation (24). However, neither our cohort nor other studies involving two devices reported observing DRT.

Congruent with the findings of the previous case series and consistent with the findings of the two preceding studies on Watchman devices (6, 10), our investigation demonstrated that the implantation of two Amplatzer devices, including use of the double-sheath approach, is technically feasible and associated with promising short-term safety. None of the patients in our cohort experienced relevant peri-interventional complications, and complete sealing of the LAA at the end of procedure was successfully achieved in all 10 cases immediately following the intervention. Importantly, no thromboembolic events occurred throughout the study period.

Other post-interventional complications, including stroke, vascular access-related complications, and pericardial effusions, are valid concerns following LAAC, with pericardial effusion being among the most frequently reported adverse events (12, 18, 21). In the present study, one patient (10%) developed a non-clinically significant pericardial effusion that resolved without sequelae. Pericarditis occurred in three patients (30%). In two cases—presenting 7 and 10 days after implantation—a device-related mechanism was considered highly probable. The third case, occurring 11 months after implantation, was deemed less likely to be causally related to the device. Previous reports of dual Watchman devices described a single case of relevant pericardial effusion requiring pericardiocentesis, but no cases of pericarditis (6, 10). Although all episodes in our cohort were hemodynamically stable and responded to anti-inflammatory therapy without hospitalization or sequelae, the incidence observed appears elevated compared with published experience for single-device, endocardial LAAC. For example, in a large National Inpatient Sample analysis of over 38,000 Watchman procedures, in-hospital pericarditis was recorded in only 0.2% of patients, while overall pericardial complications occurred in 3.8% (25). By contrast, epicardial LAA ligation with the LARIAT system has been associated with early post-procedural pericarditis rates of approximately 4%, which were reduced to approximately 1.6% with systematic colchicine prophylaxis (26). Comparative studies of the Amulet and Watchman devices suggest that the Amulet device may carry a higher risk of post-procedural pericardial effusion (12, 18, 21, 27). However, data on pericarditis following LAAC remain scarce, with no well-defined studies systematically evaluating its incidence (28, 29). The pathophysiology of post-LAAC pericarditis is incompletely understood, but the most plausible mechanism is post-cardiac injury syndrome, triggered by myocardial or pericardial injury with subsequent release of cardiac antigens (30). Local irritation from exposed device components—particularly the stabilizing hooks of the Amulet device contacting the LAA wall—represents a likely contributor. Other potential mechanisms include device friction, excessive tension, or puncture-related trauma. In our series, both early pericarditis episodes occurred in patients treated with two Amulet devices using a double-sheath technique, whereas the delayed case arose after a single-sheath implantation of an ACP and AVP. This pattern raises the hypothesis that the combination of a disc-and-lobe design and overlapping devices may amplify local mechanical irritation or microperforation of the LAA, thereby predisposing to an exaggerated inflammatory response. The double-sheath technique, requiring two venous accesses and two transseptal punctures, increases the risk for pericardial effusion but not for pericarditis. While our sample size is too small to define an absolute excess risk or to disentangle the respective contributions of device design, dual-device implantation, and double-sheath access, the high proportion of pericarditis observed here underscores the need for vigilant post-procedural monitoring, systematic reporting of this endpoint in future LAAC studies, and prospective evaluation of targeted preventive strategies (e.g., short-term colchicine) in anatomically complex cases in which a dual-device strategy is contemplated.

## 5 Limitations

The results of this study should be interpreted in light of several limitations. First, the observational and retrospective design, together with the small sample size of only 10 patients, inherently limits the external validity and generalizability of the findings. Accordingly, statistical comparisons between the dual- and single-device groups should be considered descriptive and hypothesis-generating rather than inferential, given the very small number of patients in the dual-device cohort and the pronounced imbalance between the groups.

Second, no direct comparison between single- and dual-device approaches was performed regarding clinical outcomes such as all-cause mortality, cardiovascular events, or bleeding complications, leaving open the question of which strategy provides the most favorable results. It should be emphasized, however, that the dual-device approach was reserved for highly complex LAA anatomies in which a single device would not have achieved complete occlusion. In general, a single-device strategy is preferred when adequate sealing can be obtained, whereas the dual-device approach should be considered a bail-out option for anatomically challenging cases.

Third, only Amplatzer devices were used—albeit of different generations—making it impossible to determine which device may be most suitable for large or multilobed LAA anatomies. The advent of newer and larger occluders, such as the LAmbre or Watchman FLX Pro 40 mm, may provide alternative solutions for such anatomies in the future.

Fourth, this cohort consisted exclusively of male patients, which limits the extrapolation of the findings to the female population. This limitation is clinically relevant, as Amplatzer devices have been associated with an increased incidence of pericardial effusion, and recent evidence suggests that female sex may constitute an independent risk factor for this complication (12). Furthermore, potential sex-specific differences in procedural or clinical outcomes following dual-device implantation—particularly regarding device tolerance, the development of DRT, and the efficacy of thromboembolic event prevention—remain to be elucidated.

Fifth, the follow-up duration was limited to 1 year, precluding conclusions regarding the long-term safety and efficacy of this approach, particularly in relation to thromboembolic prevention.

Sixth, postoperative antithrombotic management was heterogeneous, preventing firm conclusions about the optimal pharmacologic regimen in this specific setting. This question remains unresolved even among patients undergoing single-device implantation.

Seventh, all dual-device implantations were performed concomitantly; therefore, it remains unknown whether a staged procedure might be advantageous in selected complex anatomies.

Eighth, TEE guidance was used in only five procedures, limiting the precision of procedural assessment and the ability to reliably exclude PDLs at completion.

## 6 Conclusion

Concomitant implantation of two Amplatzer devices in patients with multilobed LAA anatomies appears to be technically feasible and demonstrates promising short-term safety. The incidence of post-procedural pericarditis may be higher compared to single-device implantation. Our findings thus indicate that multilobed anatomy no longer represents an exclusion criterion for percutaneous closure with reliable complete sealing. Larger studies with longer follow-up periods are required to confirm these findings.

## Data Availability

The original contributions presented in the study are included in the article/Supplementary Material; further inquiries can be directed to the corresponding author.
